# The Increased Activity of Liver Lysosomal Lipase in Nonalcoholic Fatty Liver Disease Contributes to the Development of Hepatic Insulin Resistance

**DOI:** 10.1155/2012/135723

**Published:** 2011-08-23

**Authors:** Monika Cahova, Helena Dankova, Eliska Palenickova, Zuzana Papackova, Radko Komers, Jana Zdychova, Eva Sticova, Ludmila Kazdova

**Affiliations:** ^1^Department of Metabolism and Diabetes, Institute for Clinical and Experimental Medicine, Videnska 1958/9, 14021 Prague 4, Czech Republic; ^2^Diabetes Center, Institute for Clinical and Experimental Medicine, 14021 Prague 4, Czech Republic; ^3^Division of Nephrology and Hypertension, Oregon Health and Science University, Portland, OR 97239-3098, USA; ^4^Department of Medicinal and Clinical Chemistry, University of Heidelberg, 69117 Heidelberg, Germany; ^5^Laboratory of Experimental Hepatology, Institute for Clinical and Experimental Medicine, 14021 Prague 4, Czech Republic

## Abstract

We tested the hypothesis that TAG accumulation in the liver induced by short-term high-fat diet (HFD) in rats leads to the dysregulation of endogenous TAG degradation by lysosomal lipase (LIPA) via lysosomal pathway and is causally linked with the onset of hepatic insulin resistance. We found that LIPA could be translocated between qualitatively different depots (light and dense lysosomes). In contrast to dense lysosomal fraction, LIPA associated with light lysosomes exhibits high activity on both intracellular TAG and exogenous substrate and prandial- or diet-dependent regulation. On standard diet, LIPA activity was upregulated in fasted and downregulated in fed animals. In the HFD group, we demonstrated an increased TAG content, elevated LIPA activity, enhanced production of diacylglycerol, and the abolishment of prandial-dependent LIPA regulation in light lysosomal fraction. The impairment of insulin signalling and increased activation of PKC**ε** was found in liver of HFD-fed animals. Lipolysis of intracellular TAG, mediated by LIPA, is increased in steatosis probably due to the enhanced formation of phagolysosomes. Consequent overproduction of diacylglycerol may represent the causal link between HFD-induced hepatic TAG accumulation and hepatic insulin resistance via PKC**ε** activation.

## 1. Introduction

NAFLD (nonalcoholic fatty liver disease) is often associated with insulin resistance (IR) and type 2 diabetes [1]. High-fat diet-induced liver triacylglycerol (TAG) accumulation results in the hepatic IR even after three days of administration and without significant impairment of insulin-mediated peripheral glucose disposal [2]. However, the mechanism by which hepatic fat accumulation might lead to the hepatic insulin resistance is far from being clearly understood [3]. The TAG metabolism in the liver is subject to a highly sensitive regulation in order to fulfil the actual needs of the organism. It has been shown that the liver is a site of continuous lipolysis of endogenous TAG and partial reesterification of released free fatty acids (FFA) back to the intracellular lipid storage pool [4]. The rate of intracellular lipolysis is 2-3 times greater than required to maintain the observed rate of TAG secretion [5]. 

Nevertheless, in spite of intensive research in this field, there are many uncertainties concerning the enzyme(s) responsible for intracellular TAG degradation. One possible candidate is lysosomal lipase (LIPA) [6]. It belongs to a group of more than 50 acid hydrolases that are characterised by low pH optimum (4.5–5). Because these enzymes require a pH range that is incompatible with the neutral cytoplasma milieu, they are sequestered in specific cytoplasmic particles termed lysosomes [7]. Due to a large variety of lysosomal enzymes (including proteases, lipases, glycosidases, and nucleases), lysosomes mediate complete breakdown of many types of molecules and confer upon this organelle its high degradative capacity [8]. Lysosomal enzymes are synthesized in endoplasmic reticulum, sequestered into specialised regions of Golgi apparatus, and bud out and detach as small vesicles called primary lysosomes [9]. Substrates can reach lysosomes via heterophagy (including exocytosis and phagocytosis), in which cargo originates at the plasma membrane or extracellularly, or via autophagy, for cargo located in the cytosol. Material designed for degradation is temporally stored in digestively inactive organelles termed phagosomes. Only after fusion of phagosome with primary lysosome and acidification of intralysosomal space could the internalized material be degraded and the degradation products released back into cytoplasm [10]. The lysosomal pathway was originally associated with removal of organelles and degradation of proteins [11]. Only recently the critical role of this pathway in metabolism and storage of intracellular lipids has been discovered [12]. Hayase and Tappel [13] showed that lysosomal lipase is capable of hydrolyzing triacylglycerols and that the dominant products of lysosomal lipase action on TAG molecule are diacylglycerol (DAG) and one molecule of fatty acid. DAG is a known activator of classic and novel isoforms of protein kinase C (PKC), and DAG concentrations have closely paralleled insulin resistance in other models [14, 15]. While PKCs, in general, have been implicated in the pathogenesis of insulin resistance in many tissues, Samuel et al. [16, 17] delineated the specific role of one particular isoform, PKC*ε*, in the development of fat-induced insulin resistance in the liver.

We hypothesized that steatosis-associated hepatic IR is causally linked with alteration of endogenous TAG degradation in NAFLD. To address this issue, the activity of LIPA and the production of TAG breakdown intermediates were determined in animals with normal insulin sensitivity and with hepatic IR induced by a two-week administration of a high-fat diet. We identified the steatosis-associated changes in the regulation of LIPA activity based on the alteration in its intracellular distribution, and we proposed the mechanism by which it can contribute to the establishment of hepatic IR.

## 2. Materials and Methods

### 2.1. Animals and Experimental Protocol

Male rats were kept in temperature-controlled room at 12 : 12 h light-dark cycle. Animals had free access to drinking water and diet if not stated otherwise. All experiments were performed in agreement with the Animal Protection Law of the Czech Republic 311/1997 which is in compliance with Principles of Laboratory Animal Care (NIH publication no. 85-23, revised 1985) and were approved by the ethical committee of the Institute for Clinical and Experimental Medicine. Starting at age 3 months (b.wt. 300 ± 20 g), all animals were fed either HFD (70 cal% as saturated fat, 20 cal% as protein, and 10 cal% as carbohydrate) or standard laboratory chow diet (SD) for 2 weeks. The groups labelled SD fed or HFD fed had free access to the diet until decapitation (10–11 am), and the groups designated as SD fasted or HFD fasted were deprived of food for the last 24 hours. Glucose tolerance was determined as the rate of disappearance of glucose from circulation after a single dose of glucose (3 g/kg b.wt.) administered intragastrically to overnight-fasted animals. 

### 2.2. Preparation of Lysosomal and Phagolysosomal Fractions

The lysosomes and phagolysosomes represent a heterogeneous population of organelles. 20% (wt/vol) homogenate was prepared by homogenization of liver tissue in 0.25 M sucrose; 0.001 M EDTA pH  = 7.4; heparin 7 IU/m, 1 mM PMSF, leupeptin 10 *μ*g/mL, and aprotinin 10 *μ*g/mL by Teflon pestle homogenizer. The crude impurities were removed by brief centrifugation at 850 g. The fat cake was removed carefully in order to prevent contamination of liquid fraction. An aliquot of the homogenate was kept at 4°C until lipase assay (maximum 2 hour), the rest was centrifuged for 10 000 g 20 min 4°C, and the resulting pellet and supernatant were separated. The supernatant contains preferentially the less dense lysosomes with higher TAG content (light lysosomes), and the pellet is formed by more dense particles (dense lysosomes). 

### 2.3. Assay of Triacylglycerol Lipase Activity on Exogenous Substrate

The optimal conditions for the lipase assay (substrate concentration, reaction temperature, and linear range of the assay) were determined in the pilot experiments. The data are provided in supplements (a–c). 4% liver homogenate or lysosomal subfractions prepared from the fresh tissue under iso-osmotic conditions were used for the assay. The reaction medium (92.5 kBq ^3^H triolein, 100 *μ*M triolein, 110 *μ*M lecithin, 0.15 M NaCl, and 0.1 M acetate buffer pH 4.5) was emulsified by sonication (Hielsler sonicator UP200S). The assay itself was performed under hypoosmotic conditions (50 mM sucrose) in order to ensure the release of the enzyme sequestered within the lysosomes. The liver homogenate or isolated fractions were incubated for 60 minutes at 30°C. The released fatty acids were extracted according to [18] and counted for radioactivity. 

### 2.4. Assay of Triglyceride Lipase Activity on Endogenous Substrate

This approach takes advantage of the coordinated changes in the intracellular localisation of LIPA and its intracellular substrate. The optimal conditions for the lipase assay were determined in the pilot experiments. The data are provided in supplements (e, f). The liver homogenate and subcellular fractions were prepared as described above under iso-osmotic conditions that prevent the disruption of lysosomes. The lysis of lysosomes was induced only after separation of fractions during the assay. 20% homogenate was mixed 1 : 1 with 0.2 M acetate buffer pH  = 4.5 and incubated for 60 min in 30°C in shaking water bath. The reaction mixture was extracted in chloroform-methanol, and phases were separated by 1 M NaCl. 

Aliquots of lower chlorophorm phase were separated for further determination of FFA and DAG content. An aliquot of chlorophorm phase was evaporated, and 100 *μ*L of Krebs-Ringer phosphate buffer (pH  = 7.6) containing 6% FFA-free BSA was added. The tubes were incubated in shaking incubator at 37°C for 2 hours. FFA concentration in final KRF/BSA solution was measured using commercially available kit. In order to check the efficiency of FFA solubilisation, the emptied tubes were washed with fresh KRB + 6%  BSA, and then 100 *μ*L of chlorophorm was added. An aliquot was separated by TLC, but no substantial traces of FFA were detected. 

### 2.5. Determination of DAG Content

This method is based on the phosphorylation of DAG in the sample to DAG-3-phosphate using *γ* ^35^-ATP followed by quantification of radioactivity in a chlorophorm extract. Lipids from liver tissue or incubation mixture were extracted in chlorophorm-methanol and an aliquot of chlorophorm phase was evaporated under the stream of nitrogen. The sample was than solubilised by sonication in detergent buffer (7.5% n-octyl-*β*-D-glucopyranoside, 5 mM cardiolipin, and 1 mM DETAPAC). Reaction buffer (50 mM imidazole/HCl, pH  = 6.6, 50 mM NaCl, 12.5 mM MgCl_2_, and 1 mM EGTA), diacylglycerol kinase, and *γ* ^35^-ATP were added and incubated 30 min in 25°C. Lipids were extracted into chlorophorm-methanol, phases were separated with 1% HCLO_4_, and the exact volume of lower chlorophorm phase was determined. An aliquot was evaporated, resolved in 5% chlorophorm-methanol, and separated by TLC. Individual populations of lipids were visualised by iodine vapours, the bands corresponding to DAG were scraped off, and the radioactivity was determined by scintillation counting. 

### 2.6. Incubation of Liver Slices In Vitro

The production of *β*-hydroxybutyrate from liver slices *in vitro *was measured in the absence of exogenous FFA. Liver slices (width approximately 1 mm) were quickly dissected and incubated for 2 hours in Krebs Ringer bicarbonate buffer with 5 mmol/L glucose, 2% bovine serum albumin, gaseous phase 95% O_2_, and 5% CO_2_. All incubations were carried out at 37°C in sealed vials in a shaking water bath. The aliquots of the incubation medium were stored frozen until the further analysis.

### 2.7. Electrophoretic Separation and Immunodetection

The homogenate, light lysosomal fraction, and dense lysosomal fraction prepared as described above were used for the assessment of LIPA protein content. A separate group of rats were used to assess the impact of hepatic fat accumulation on the insulin signalling pathway. The animals were either deprived of food for 24 hours (fasted) or had free access to food, and insulin (6 U/kg i.p.) was administered 30 min prior decapitation (fed + insulin). Liver samples (200 mg) were harvested *in situ* and stored in liquid nitrogen until further utilization. The homogenate was prepared by Ultra-Turax homogenizer (IKA Worke, Staufen, Germany) in homogenization buffer (150 mM NaCl, 2 mM EDTA, 50 mM TRIS, 20 mM glycerolphosphate, 1 mM Na_3_VO_4_, 2 mM sodium pyrophosphate, 1 mM PMSF, leupeptin 10 *μ*g/mL, and aprotinin 10 *μ*g/mL). The homogenate was used for determination of mTOR and Akt phosphorylation. The proteins were separated by electrophoretic separation under denaturing conditions and electroblotted onto PVDF membranes. The level of phosphorylation of Akt and mTOR kinases was assessed by immunodetection using specific phospho-Akt (Ser473) antibody and phospho-mTOR (Ser2448) antibody, respectively. The total expression of Akt and mTOR protein was determined on the same membrane after striping and reblotting using specific antibodies. All these antibodies were purchased from Cell Signalling Technology, (Boston, MA). The immunodetection of LIPA protein was performed using mouse monoclonal (9G7F12) antibody to lysosomal acid lipase (Abcam, Cambridge, UK). The loading control was performed using rabbit polyclonal antibody to beta actin (Abcam, Cambridge, UK). The bands were visualized using ECL and quantified using FUJI LAS-3000 imager (FUJI FILM, Japan) and Quantity One software (Biorad, Hercules, CA). 

### 2.8. PKC Membrane Translocation

The liver homogenate was prepared as described above. The total membrane and cytosolic fractions were prepared by centrifugation of the homogenate at 100 000 g. Solubilisation of membrane fraction was carried out in 1% Triton X-100, 0.1% SDS, and 0.5% deoxycholate. After electrophoretic separation and blotting, the PKC*ε* was detected using anti-PKC*ε* antibody (Sigma, St. Louis, USA). PKC translocation was expressed as the ratio of arbitrary units of membrane bands over the cytosol bands.

### 2.9. Biochemical Analysis

TAG content in liver homogenate or phagolysosomal fraction was determined after the extraction according to Folch et al. [19]. The glycogen content was determined in fat-free dry mass after hydrolysis in 30% KOH and expressed as a glucose equivalent (*μ*moles per g dry weight).

FFA, insulin, TAG and glucose serum content, and *β*-hydroxybutyrate production were determined using commercially available kits (FFA: FFA half microtest, Roche Diagnostics GmbH Mannheim, Germany; triglycerides and glucose: Pliva-Lachema, Brno CR; insulin: Mercodia, Uppsala, Sweden; *β*-hydroxybutyrate: RanBut, RANDOX Crumlin, UK). 

### 2.10. Statistical Analysis

Data are presented as mean  ± SEM. Statistical analysis was performed using Kruskal-Wallis test with multiple comparisons (*n* = 5–7). Differences were considered statistically significant at the level of *P* < 0.05.

## 3. Results

### 3.1. The Effect of HFD on Physical and Metabolic Parameters

The two-week period of HFD resulted in higher body weight and increased fat accumulation determined as epididymal fat pad : body weight ratio (Table 1). The impairment of glucose metabolism was indicated by increased fasting glycemia, increased fasting insulinemia, and impaired glucose tolerance measured by oral glucose tolerance test and expressed as AUC_1–180 min _. The alterations in glucose metabolism were not accompanied by dyslipidemia. Serum *β*-hydroxybutyrate concentration was significantly elevated in both HFD-fed as well as HFD-fasted group compared to corresponding SD groups what indicates increased utilisation of fatty acids for ketogenesis in the liver. Short-term HFD administration did not alter ALT and AST serum concentrations. 

As expected, compared to the SD group, the HFD-administered animals accumulated increased amount of TAG (fasted: 14.6 ± 1.4 versus 3.2 ± 0.2; *P* < 0.001; fed: 16.2 ± 2.5 versus 2.9 ± 0.2 *μ*mol/g; *P* < 0.001) and DAG (fasted: 138 ± 17 versus 83 ± 12; *P* < 0.01; fed: 145 ± 19 versus 53 ± 9 nmol/g; *P* < 0.001) in the liver. The insulin-stimulated increase of glycogen content in liver was lower in HFD compared to SD animals (fasted: 27 ± 7 versus 41 ± 9 n.s.; fed: 123 ± 10 versus 261 ± 15 *μ*mol/g; *P* < 0.001).

### 3.2. The Effect of HFD on Lysosomal Lipase Activity

**Figure 1:**
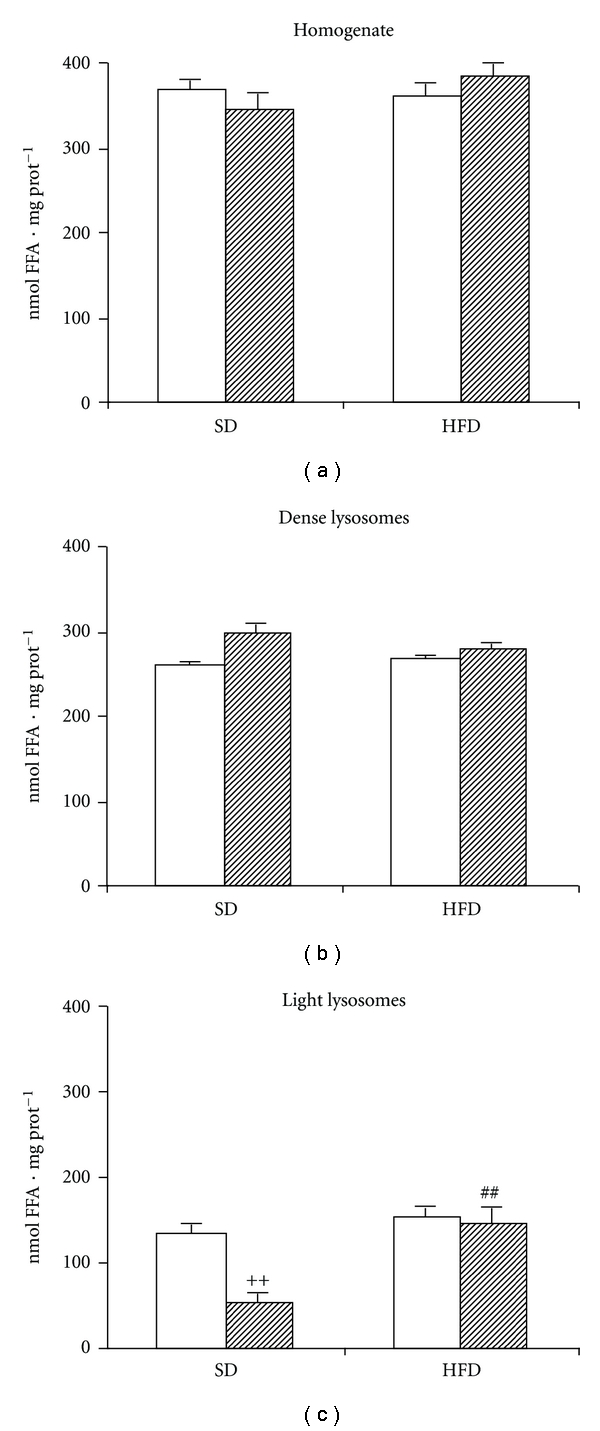
The effect of HFD on the LIPA activity measured as FFA release from artificial substrate (^3^H-triolein). (a) Homogenate; (b) dense lysosomes; (c) light lysosomes. The lipase activity was measured as the release of fatty acids at pH  = 4.5 from ^3^H-triolein. Open bars = fasted animals; hatched bars = fed animals. ^++^ *P* < 0.01 fed versus fasted; ^##^ *P* < 0.01 HFD fed versus SD fed.

In order to determine maximal LIPA activity in liver homogenate and in particular lysosomal subpopulations, we employed emulsified ^3^H-labeled triolein as a substrate. In this experimental setting, the substrate is present in excess, and the only limiting factor is the amount of enzyme. The total LIPA activity measured in the whole homogenate was not affected either by prandial status (fasted or fed state) or diet intervention (SD or HFD) (Figure 1(a)). Similar results were observed in the fraction of dense lysosomes that represent primary lysosomes (Figure 1(b)). On ^3^H-triolein as a substrate, we found most of total LIPA activity in this fraction. The LIPA activity determined in light lysosomes represents only minor portion of total activity, but unlike homogenate or dense lysosomes, it responds to different metabolic states (Figure 1(c)). In the SD group, it is elevated in fasting and depressed in fed state. HFD feeding abolished the prandial regulation of LIPA activity especially due to its upregulation in fed state. 

**Figure 2:**
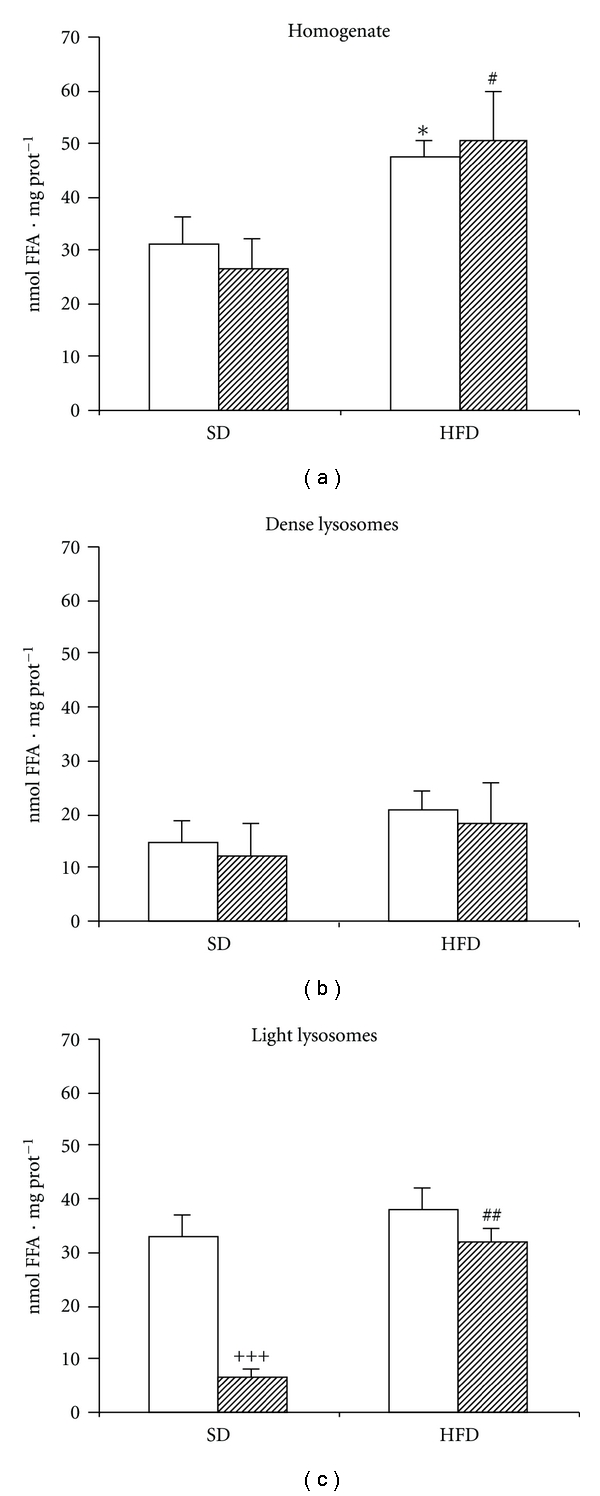
The effect of HFD on the LIPA activity measured as FFA release from endogenous TAG. (a) Homogenate; (b) dense lysosomes; (c) light lysosomes. 10% liver homogenate, light or dense lysosomal fraction were incubated 60 min at pH  = 4.5. At the end of incubation, the released FFAs were quantified as described in Section 2. The graph shows the difference between FFA concentration in the sample at the beginning and at the end of the incubation. Open bars = fasted animal; hatched bars = fed animals. ^+++^ *P* < 0.001 fed versus fasted; *P < 0.05 HFD fasted versus SD fasted; ^#^ *P* < 0.05, ^##^ *P* < 0.01 HFD fed versus SD fed.

A separate set of experiment was designed in order to evaluate the contribution of LIPA associated with dense and light lysosomes to the degradation of intracellular TAG. In this experimental design, the intracellular TAGs contained in particular fraction are the only source of substrate, and the intensity of lipolysis depends not only on the amount of enzyme but also on the amount of substrate available in the sample (Figures 2(a), 2(b), and 2(c)). Compared with the same experiments carried on ^3^H-triolein, we found two differences. First, HFD administration led to a significant increase of total LIPA activity measured in homogenate. Second, after separation of lysosomal subpopulations, LIPA activity associated with light lysosomes was higher than those associated with dense lysosomes. This observation could be explained by the previous “*in vivo”* translocation of both the substrate (TAG droplets) and the enzyme (LIPA) into light lysosomal fraction (phagolysosomes). In accordance with this presumption, we found higher TAG content in phagolysosomal fraction in HFD compared with SD group (fasted: 2.3 ± 0.2 versus 4.1 ± 0.7; fed: 1.02 ± 0.3 versus 5.6 ± 0.48 *μ*mol·mg prot^−1^). Similarly with the results obtained on ^3^H-triolein, the effect of fasting was manifested only in SD group and only in light lysosomal fraction. HFD feeding resulted into the elevation of LIPA activity in light lysosomes and into the abolishment of prandial regulation. Taken together, our results indicate that in the liver most of the enzyme is present in inactive form in dense (primary) lysosomes, and the physiologically active portion of the enzyme could be determined in light lysosomal fraction.

### 3.3. The Effect of HFD on Lysosomal Lipase Protein Distribution

**Figure 3:**
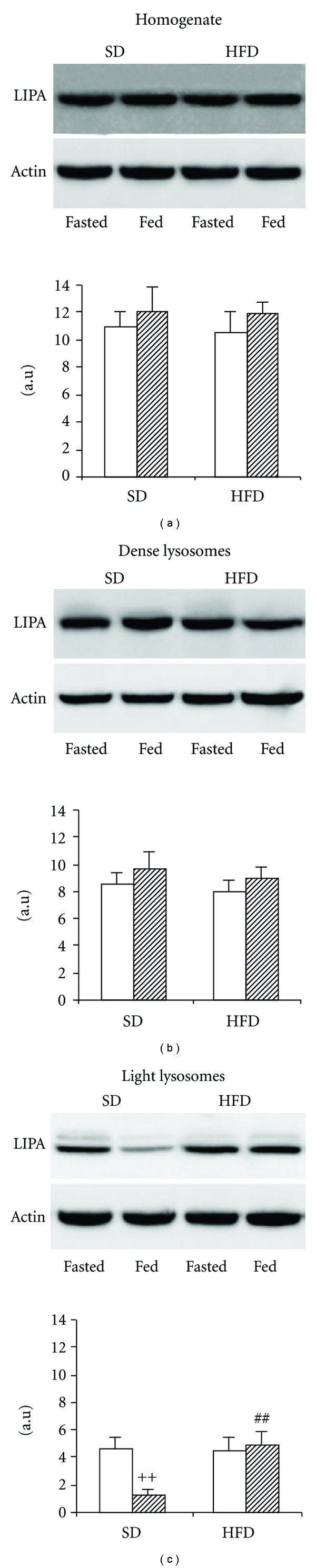
The effect of HFD on the LIPA protein expression. (a) Homogenate; (b) dense lysosomes; (c) light lysosomes. Representative Western blots are shown above each graph (f  = fasted, F  = fed). The results are expressed as arbitrary units after normalisation to the actin expression (loading control). Open bars  = fasted animals; hatched bars  = fed animals. ^++^ *P* < 0.01 fed versus fasted; ^##^ *P* < 0.01 HFD fed versus SD fed.

In order to distinguish whether the higher LIPA activity found in the light lysosomal fraction in HFD group is consequent to the increased amount of enzyme in this fraction or only to the increased availability of the substrate, we determined the amount of LIPA protein in liver homogenate and in particular fractions. We found that the LIPA protein content in homogenate (Figure 3(a)) and dense lysosomal fraction (Figure 3(b)) is similar in both fasted and fed animals and that it is not affected by short-term HFD administration. In contrast to these findings, the abundance of LIPA protein in light lysosomal fraction is lower than in homogenate or dense lysosomes, but it varies according to several factors (Figure 3(c)). In SD group, it strongly depends on prandial status. In SD-fasted rats, LIPA protein abundance in this fraction is significantly higher compared with their fed counterparts. Short-term HFD diet has no effect on the content of LIPA protein in fasted animals, but it significantly increases its amount in the fed ones. Consequently, the prandial-dependent regulation is completely abolished in HFD group.

### 3.4. Diacylglycerol Production in Incubated Liver Homogenate

**Figure 4:**
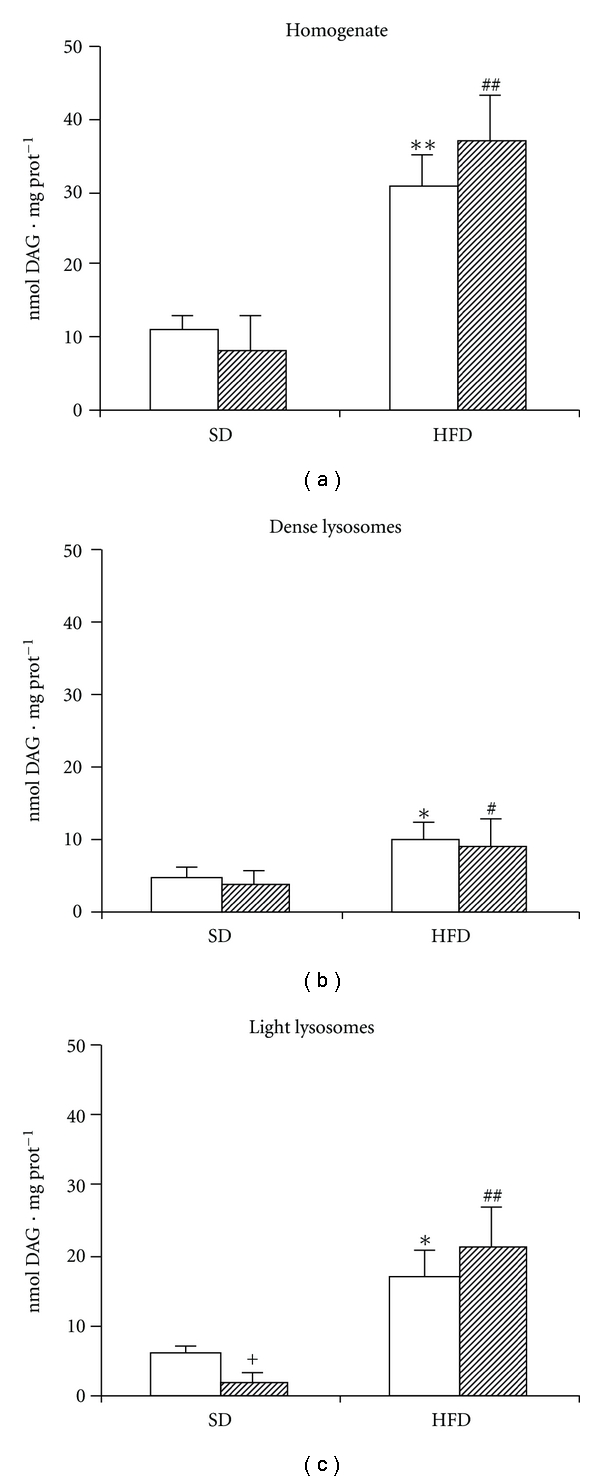
The effect of HFD on the DAG production from intracellular TAG *in vitro. *(a) Homogenates; (b) dense lysosomes; (c) light lysosomes. 10% liver homogenate, dense lysosomal or light lysosomal fractions were incubated 60 min at pH  = 4.5. At the end of incubation, DAG was extracted into chlorophorm-methanol and quantified as described in Section 2. The graph shows the difference between DAG concentration in the sample at the beginning and at the end of the incubation. Open bars  = fasted animals; hatched bars  = fed animals. ^+^ *P* < 0.05 fed versus fasted; *P < 0.05, **P < 0.01 HFD fasted versus SD fasted; ^#^ *P* < 0.05, ^##^ *P* < 0.01 HFD fed versus SD fed.

DAG is one of the major products of LIPA action on TAG molecule as this enzyme has lower affinity to DAG or monoacylglycerol compared with its affinity to TAG [13]. In our experimental conditions (incubation of liver homogenate or isolated fraction in pH  = 4.5), DAG could not be further utilised for TAG biosynthesis, and the difference in DAG concentrations at the end and at the beginning of incubation represents the net DAG production from TAG degradation. Nevertheless, we cannot exclude some degradation of DAG by lysosomal carboxylesterases. In homogenate, DAG production in SD group was significantly lower compared with those in HFD, and it was prandial dependent, that is, elevated in fasting and downregulated in fed state. In HFD group, a significant DAG production was detected in both fasted and fed animals (Figure 4(a)). The stimulatory effect of HFD on DAG production was found in both dense (Figure 4(b)) and light (Figure 4(c)) lysosomes. In HFD group, approximately 60% of DAG formation occurred in light lysosomal fraction, and in contrast to the SD group, it was independent of prandial status.

### 3.5. The Effect of HFD on Ketogenesis In Vitro

**Figure 5:**
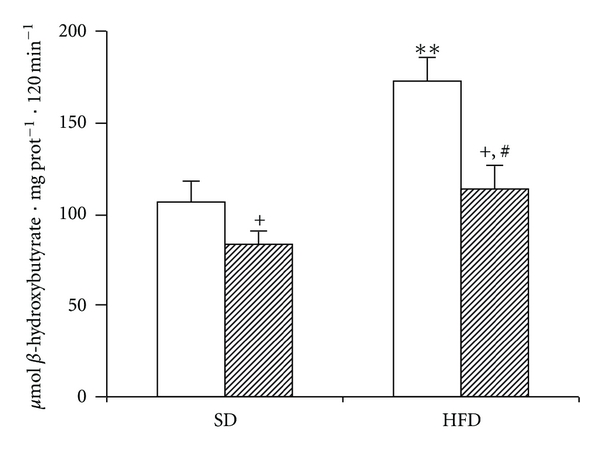
The effect of HFD on *β*-hydroxybutyrate production from liver slices *in vitro.* Liver slices were incubated in oxygenated KRB without exogenous fatty acids. Open bars  = fasted animals; hatched bars  = fed animals. ^+^ *P* < 0.05 fed versus fasted; **P < 0.01 HFD versus SD fasted; ^#^ *P* < 0.05; ^#^ *P* < 0.05 HFD fed versus SD fed.

LIPA is expressed not only in hepatocytes but also in many other cell types including Kupffer cells present in the liver. In order to address the issue whether the above-mentioned changes in LIPA activity could be ascribed to hepatocytes, we measured ketone bodies production from liver slices *in vitro* (Figure 5). Ketogenesis is the metabolic pathway occurring exclusively in hepatocytes and tightly reflects the intracellular TAG metabolism. We found an elevated ketogenesis due to the HFD administration what under these experimental set up implicates the accentuation of TAG hydrolysis. When liver slices were incubated in the absence of exogenous FFA, HFD-fasted group exhibited significantly higher *β*-hydroxybutyrate production compared with SD fasted. A similar trend was found also in fed animals.

### 3.6. The Effect of HFD on Key Components of Insulin Signalling Pathway

**Figure 6:**
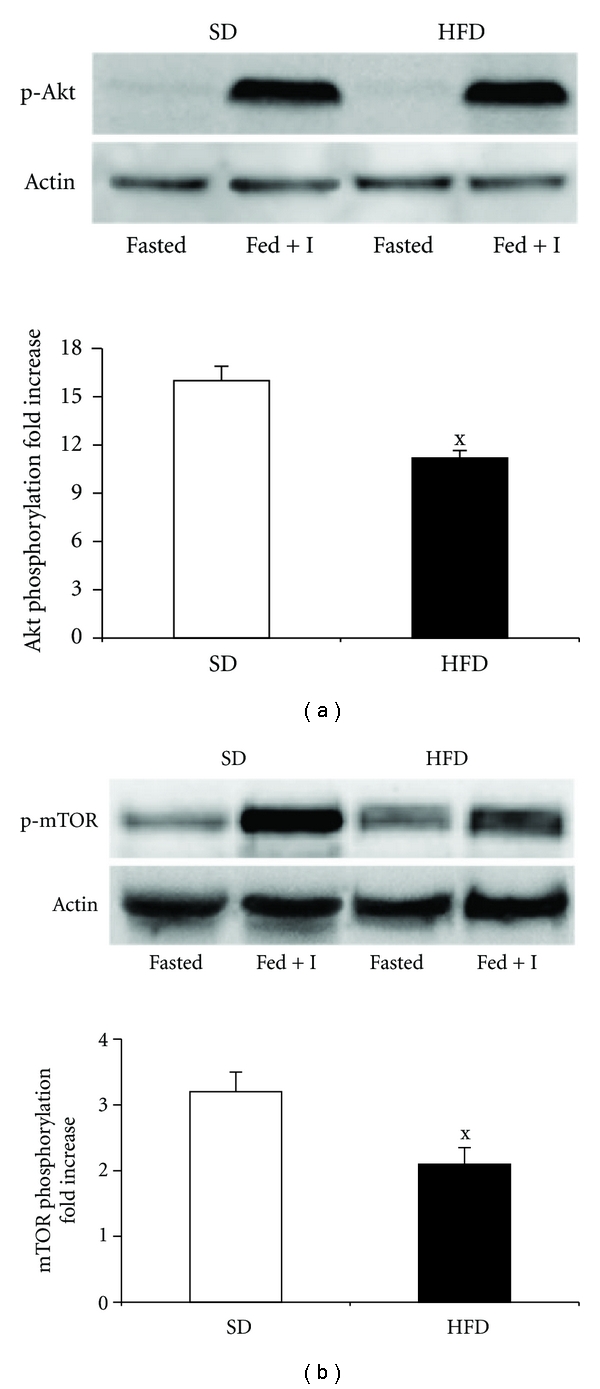
Alterations of insulin signalling cascade associated with hepatic fat accumulation. All results are expressed as a fold increase in the insulin-stimulated state relative to the basal state. Representative Western blots are shown above each graph. (a) Fold increase in Akt (Ser473) phosphorylation, (b) fold increase in mTOR (Ser2448) phosphorylation. The basal level of protein phosphorylation was determined in the homogenate prepared from the liver of 24 hours fasted animals. The effect of insulin was determined in identically processed samples from animals which had free access to food and 40 min prior to decapitation were administered insulin 6 U/kg. The total protein (Akt or mTOR) expression was determined after striping the membrane and reblotting with anti-Akt or anti-mTOR antibody. Values represent means ± S.E.M. of 7 animals. ^x^ *P* < 0.05.

To determine the effect of HFD on hepatic insulin sensitivity, the activation of key components of insulin signalling pathway was measured by immunodetection of their phosphorylation status. As shown in Figure 6(a), the insulin-stimulated phosphorylation of Akt kinase was significantly impaired in HFD-compared to SD group. Similar results were obtained for mTOR (Figure 6(b)) suggesting an impairment of insulin signal transduction.

### 3.7. The Effect of HFD on PKC*ε* Activity

**Figure 7:**
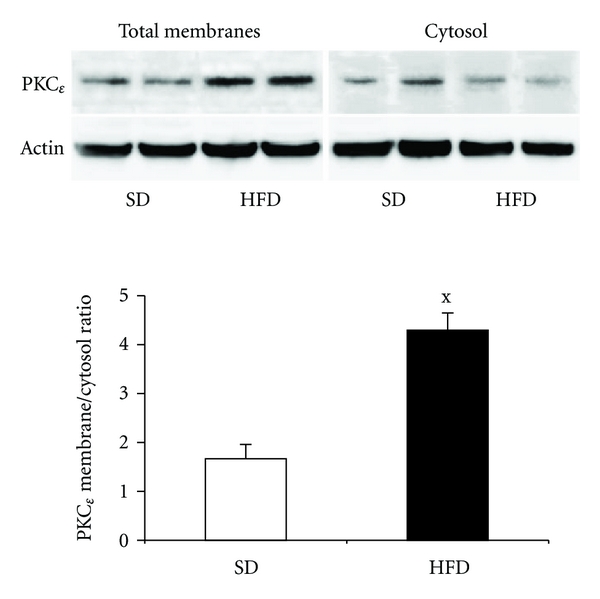
The effect of hepatic fat accumulation on PKC*ε* activation. Representative Western blot is shown in the upper part of the figure; TM, total membrane fraction, C, cytosol fraction. The PKC*ε* membrane to cytosol ratio is shown in the graph. The relative densities of the bands in the membrane fraction were compared with corresponding ones in cytosol fraction in order to obtain the measurable parameter of activation. Values represent means  ± S.E.M. of 7 animals. ^x^ *P* < 0.05.

Determination of the relative abundance of the particular PKC isoform in the membrane and cytosol fractions reflects PKC*ε* activation. An increase in the membrane to cytosol fraction ratio was used as an indicator of PKC*ε* activation. As shown in Figure 7, PKC*ε* was significantly activated in the liver of the HFD-administered animals.

## 4. Discussion

In the present study, we provide evidence that in steatosis the increased degradation of TAG mediated by LIPA and associated with the increased production of DAG may be one of the mechanisms determining the rapid onset of hepatic IR. Our hypothesis is based on following findings. First, alterations in LIPA activity associated with different metabolic states are based on prandial-dependent translocation of the enzyme from the inactive pool of dense lysosomes into light lysosomal fraction, and it is upregulated in fasting and downregulated in the fed state. After short-term HFD administration, this prandial-dependent regulation of LIPA activity is abolished. The fed state-associated downregulation of LIPA activity is impaired, and the portion of the active enzyme is permanently increased. These changes were demonstrated on both endogenous TAG and exogenous substrate (emulsified ^3^H-triolein). Second, in steatosis, the production of TAG degradation intermediates, FFA and DAG, by lysosomal lipase was significantly elevated. Finally, we proved an increased PKC*ε* activation together with the defects in the insulin-signalling cascade in the fatty liver. Taken together, these data indicate that the enhanced activity of LIPA in HFD-fed animals and following overproduction of PKC*ε* activator DAG contribute to the establishment of HFD-induced IR. We have previously shown that LIPA is involved in the degradation of intracellular TAG in the liver [20]. The essential role of LIPA for hydrolysis of TAG is supported by findings of Du et al. [21] who reported that LIPA knock-out mice (*Lipa* ^−^/^−^) exhibited progressive hepatosplenomegaly and massive TAG accumulation in the liver. 

Our data indicate that the principal factor regulating the LIPA activity is not the total amount of the enzyme itself but rather its intracellular localisation. We did not find any significant differences in LIPA mRNA expression in response to either fasting or diet intervention (not shown), and in accordance with this, we did not find any difference in total LIPA protein content determined in the whole homogenate. According to Seglen and Solheim [22], active phagolysosomes have a lower density than the small, inactive lysosomes, allowing their separation by differential centrifugation. Based on this observation, we separated the total lysosomes into two subpopulations according to their density. We expected that the active lysosomes containing the TAG substrate would remain in the less dense fraction (light lysosomes), while the inactive lysosomes would sediment (dense lysosomes). In our experimental setting, the effect of fasting or HFD was manifested predominantly in light lysosomal fraction what supports the physiological relevance of this methodology. In SD group, we observed a significant prandial-dependent regulation, LIPA activity being upregulated in fasted and downregulated in fed animals. HFD feeding was associated with a significant elevation of LIPA protein content and LIPA activity in light lysosomal fraction particularly in fed animals and consequently with the abolishment of prandial-dependent regulation of LIPA activity. Similar trends were observed on both exo- and endogenous substrates. The changes in LIPA activity were reflected by the corresponding changes in LIPA protein content in light lysosomal fraction. The interesting conclusions come from the comparison of LIPA activity in the light and dense lysosomal fractions determined on either ^3^H-triolein or intracellular TAG. The activity measured on ^3^H-triolein depends only on the amount of the enzyme present in the particular fraction as the substrate is available in excess. In contrast, when intracellular TAGs are the only source of substrate, the activity in particular fractions depends on the coordinated translocation of the enzyme and the substrate. The main difference in LIPA activity determined by these two approaches was found in the distribution of LIPA activity among dense and light lysosomal fractions. In dense lysosomes, we found high LIPA activity on ^3^H-triolein but only low LIPA activity on intracellular TAG. This difference indicates that dense lysosomal fraction contains an enzyme that is not active in physiological situation but that could be activated after addition of the arteficial substrate. The LIPA activity determined in light lysosomes represented the bulk of total LIPA activity on intracellular TAG substrate but only minor portion of total activity determined on ^3^H-triolein. It is possible to speculate that the LIPA activity on endogenous substrate quantitatively reflects the formation of activated lysosomes, that is particles containing both the substrate and the enzyme. Taken together, these data indicate that LIPA associated with light lysosomes represents the physiologically active enzyme.

We suppose that in NAFLD, characterised by high TAG intracellular content, one of the factors determining the phagolysosomal formation may be the substrate availability itself. The increased amount of intracellular lipid droplets in steatosis could promote the phagolysosome formation and stimulate the lysosomal lipolysis. Only recently, Singh et al. [23] described direct involvement of autophagy and lysosomal pathway in the degradation of intracellular lipid droplets in the liver. They found that lipid droplets can enter the autophagic degradation pathway in the same manner as proteins and damaged organelles via formation of autophago(lipo)somes that further fuses with primary lysosomes. As the only known lysosomal enzyme with lipolytic activity is LIPA, we believe that our results are in accordance with findings of Singh et al..

 The ketone body formation tightly reflects the liver lipid metabolism. Debeer et al. [24] demonstrated that both ketogenesis and FFA oxidation are a particularly good markers of lysosomal TAG degradation. We observed higher *β*-hydroxybutyrate concentration in serum and higher ketone body production from isolated liver slices in the absence of exogenous fatty acids in HFD group. We conclude that these data provide indirect evidence that confirms the stimulatory effect of short-term HFD on lysosomal lipolysis. 

Concomitantly occurring stimulation of lipolysis and the accumulation of endogenous TAG after HFD administration seem to be contradictory. However, in hepatocytes, a significant portion of FFA released from intracellular TAG (approximately 70%) is reesterified back [25]. HFD impairs VLDL secretion [26], and most of FFA reenter the intracellular storage pool. We have previously reported that DGAT-1 expression is increased in fatty liver what indicates enhanced esterification of fatty acids and may result in the intensification of lipolytic/reesterification cycle in hepatocytes [20]. The increased lipolysis thus does not result in decreased TAG content but only in higher TAG turnover.

In the liver, PKC*ε*, member of novel PKCs subfamily, is involved in the development of HFD-induced IR [16, 17]. Samuel et al. showed that fat-induced hepatic IR may result from activation of PKC*ε* and its downstream targets. Nevertheless, the nature of the signal that activates PKC*ε* has not been fully explained. Systemic increase in FFA serum levels, as one possible underlying factor, has not been described after HFD administration. Another candidate, 1,2-sn-DAG, is an important intracellular signalling molecule, and it is the known activator of novel PKCs isoform family [27]. The increased DAG content due to the increased flux through TAG synthetic pathway and the following PKC*ε* activation was described in skeletal muscle in HFD-administered animals [28]. However, DAG is also an intermediate in TAG degradation pathway that, in contrast to muscle, is quite active in the liver. Our findings suggest that DAG originating from the increased lipolytic activity of LIPA and accentuated TAG breakdown could act as PKC*ε* activator in fatty liver. This hypothesis is supported by the fact that in fatty liver LIPA is activated specifically in the fed state, and possible PKC*ε* activator is available during the period of insulin action. 

In conclusion, we found that short-term HFD-induced TAG accumulation in the liver is associated with the increased degradation of intracellular TAG by lysosomal lipase and with higher production of lipolytic products—DAG and FFA. Our findings suggest that the elevated DAG production by LIPA activated by increased supply of dietary lipids may represent the causal link between dietary fat-induced hepatic TAG accumulation and hepatic IR via the PKC*ε* activation. In the light of these findings, lysosomal lipolysis may represent a new promising therapeutic target.

## Figures and Tables

**Table 1:** The effect of HFD on physical and metabolic parameters.

	Standard diet		High-fat diet	
	Fasted	Fed	Fasted	Fed
Body weight (g)	331 ± 6.9		379 ± 10.7^x^	
Epididymal fat pad/b.w. (g/100 g)	0.9 ± 0.07	1 ± 0.06	1.4 ± 0.07*	1.4 ± 0.04^#^
Glycemia (mmol/L)	5.1 ± 0.1	7.9 ± 0.5^+^	5.9 ± 0.2*	8.1 ± 0.3^+^
Insulinemia (pmol/L)	56 ± 15	135 ± 21^+^	125 ± 10*	127 ± 18
AUC_0–180_ (mmol glucose/L)	1168 ± 26.6		1325 ± 32.9^x^	
Serum Tg (mmol/L)	0.7 ± 0.1	1.4 ± 0.08^+^	0.7 ± 0.02	1.4 ± 0.1^+^
Serum FFA (mmol/L)	0.7 ± 0.05	0.4 ± 0.02^+^	0.6 ± 0.08	0.45 ± 0.07^+^
ALT (*μ*kat/L)	1.2 ± 0.1		1.2 ± 0.1	
AST (*μ*kat/L)	4.3 ± 0.6		3.9 ± 0.3	
*β*-hydroxybutyrate (*μ*mol/L)	1.67 ± 0.05	0.05 ± 0.01^+^	3.2 ± 0.25*	0.28 ± 0.05^#,+^

Data are given as means ± SEM, *n* = 7. ^x^ *P* < 0.05 HFD versus SD group; ^+^ *P* < 0.05 fasted versus fed animals; *P < 0.05 SD- versus HFD-fasted animals; ^#^ *P* < 0.05 SD- versus HFD-fed animals.
